# Analysis of Myc-Induced Histone Modifications on Target Chromatin

**DOI:** 10.1371/journal.pone.0003650

**Published:** 2008-11-05

**Authors:** Francesca Martinato, Matteo Cesaroni, Bruno Amati, Ernesto Guccione

**Affiliations:** Department of Experimental Oncology, European Institute of Oncology (IEO), IFOM-IEO Campus, Milan, Italy; University of Arkansas for Medical Sciences, United States of America

## Abstract

The c-*myc* proto-oncogene is induced by mitogens and is a central regulator of cell growth and differentiation. The c-*myc* product, Myc, is a transcription factor that binds a multitude of genomic sites, estimated to be over 10–15% of all promoter regions. Target promoters generally pre-exist in an active or poised chromatin state that is further modified by Myc, contributing to fine transcriptional regulation (activation or repression) of the afferent gene. Among other mechanisms, Myc recruits histone acetyl-transferases to target chromatin and locally promotes hyper-acetylation of multiple lysines on histones H3 and H4, although the identity and combination of the modified lysines is unknown. Whether Myc dynamically regulates other histone modifications (or marks) at its binding sites also remains to be addressed. Here, we used quantitative chromatin immunoprecipitation (qChIP) to profile a total of 24 lysine-acetylation and -methylation marks modulated by Myc at target promoters in a human B-cell line with a regulatable c-*myc* transgene. Myc binding promoted acetylation of multiple lysines, primarily of H3K9, H3K14, H3K18, H4K5 and H4K12, but significantly also of H4K8, H4K91 and H2AK5. Dimethylation of H3K79 was also selectively induced at target promoters. A majority of target promoters showed co-induction of multiple marks - in various combinations - correlating with recruitment of the two HATs tested (Tip60 and HBO1), incorporation of the histone variant H2A.Z and transcriptional activation. Based on this and previous findings, we surmise that Myc recruits the Tip60/p400 complex to achieve a coordinated histone acetylation/exchange reaction at activated promoters. Our data are also consistent with the additive and redundant role of multiple acetylation events in transcriptional activation.

## Introduction

The fundamental unit of chromatin is the nucleosome, consisting of 146bp of DNA wrapped around an octamer of the core histones, H2A, H2B, H3 and H4 [1], [2]. Histones are highly basic globular proteins, with N-Terminal “tails” which can be heavily modified by a variety of posttranslational modifications, or histone marks [3]. Among these marks, some are associated with active chromatin (euchromatin) and/or gene transcription, such as trimethylation of histone H3 lysine 4 (H3K4me3), or acetylation of multiple lysines on histones H3 and H4 (H3ac and H4ac). Others marks are associated with inactive chromatin (heterochromatin) and transcriptional repression, such as trimethylation of H3 lysine 9 (H3K9me3) or 27 (H3K27), or of H4 lysine 20 (H4K20me3). The function of other marks still remains to be understood. A direct effect of histone modifications on promoter regions is the creation of a dynamic platform upon which the transcriptional machinery is recruited and assembled [4]. Based on this evidence, the “histone code” hypothesis suggests that histone marks offer binding sites to “reader” and “effector” proteins [5]. Indeed, many nuclear proteins contain motifs such as Bromodomains or Royal Family domains, that selectively interact with acetylated or methylated residues, respectively [6], [7]. Histone acetylation is catalyzed by a variety of histone acetyltransferase complexes (HATs), which usually target multiple lysine residues [8]. HATs function enzymatically by transferring an acetyl group from acetyl-coenzyme A (acetyl-CoA) to the å-amino group of specific lysine residues [9]. Acetylation, which neutralizes the positive charge of the lysine side-chain, is thought to weaken histone-DNA or nucleosome-nucleosome interactions, thereby destabilizing chromatin structure and allowing greater accessibility [10]. On this basis, histone acetylation has also been proposed to act cumulatively, with the number rather than exact position of the targeted residues accounting for downstream effects [11]. An alternative view is that there is specificity in the targeted residues. In yeast, for example, co-expressed genes, regulated by the same set of transcription factors acetylate the same lysine residues, which in turn signal for distinct downstream functions [12].

c-Myc is a transcription factor of the basic helix-loop-helix leucine zipper (bHLH-LZ) family, which dimerizes with another bHLH-LZ protein, Max. Myc/Max dimers specifically bind the DNA sequence CACGTG, a variant of the general CANNTG “E-box” consensus bound by bHLH proteins [13], [14], and activate transcription via this site [15], [16], [17]. Medium- and large-scale screens have revealed that Myc targets at least 10–15% of all cellular promoters in human cells [18], [19], [20], [21], [22]. Studies in *Drosophila* Kc cells [23] and mouse embryonic stem cells [24] indicate equally large numbers. Although the exact figure remains to be determined [21], [25], it appears that 60% or less of Myc-binding sites have a canonical E-box consensus motif.

Myc target-site recognition in the human genome is restricted by epigenetic mechanisms, which act upstream of sequence-specific DNA binding [25]. In particular, Myc preferentially associates with promoters enriched for euchromatic marks, including di- or tri-methylation of histone H3 lysines 4 (H3K4me2, H3K4me3) or lysine 79 (H3K79me2), as well as acetylation of lysines 9 and 18 (H3K9ac, H3K18ac). Once bound to its target promoters, Myc introduces further changes in chromatin. In particular, Myc interacts with a variety of histone modifiers, such as the HATs Tip60, GCN5/PCAF and p300/CBP [26], [27], [28], [29] or HAT-associated proteins such as TRRAP [28], [30] resulting in the recruitment of factors to chromatin and in local hyper-acetylation of histones [26], [27], [31], [32]. Consistent with this molecular mechanism, Myc is required for histone hyper-acetylation and transcriptional activation of target loci following mitogenic stimulation of rodent fibroblasts [31]. In summary, Myc targets already active or poised chromatin, inducing further chromatin modifications and contributing to the fine-tuning of gene expression in response to extra-cellular stimuli. However, the range of chromatin modifications regulated by Myc on its target loci remains unknown.

## Results

### Myc induces acetylation of several lysines on histone H3 and H4

**Figure 1:**
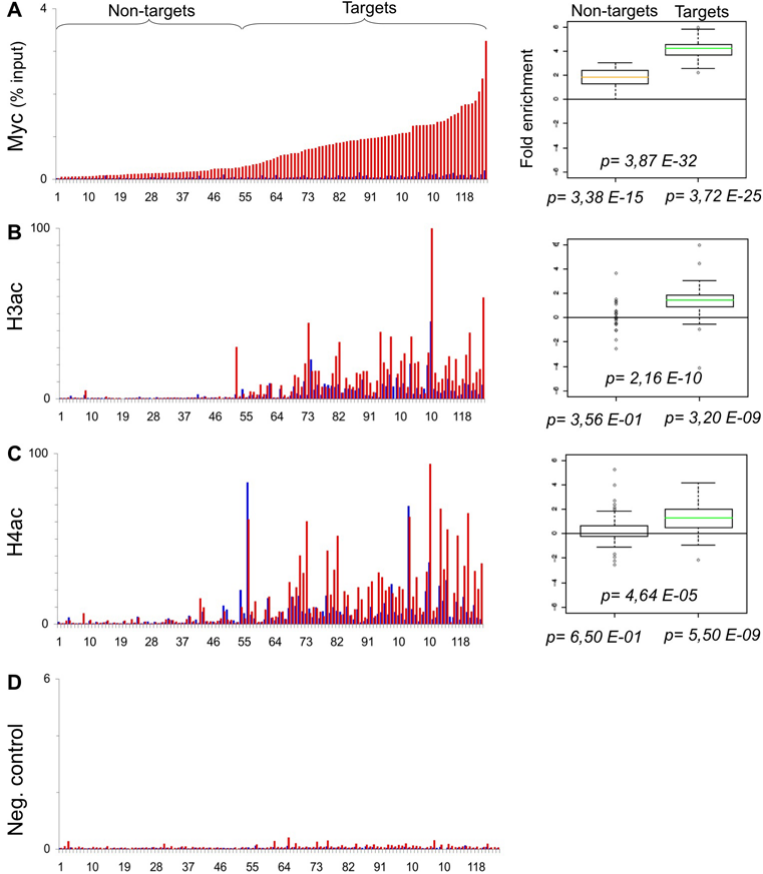
Myc induces histone H3 and H4 hyper-acetylation. A. Myc levels in two E-box containing populations are analysed in P493-6 cell line before (+Tet = blue bars) and after Myc induction (−Tet = red bars). B. Target sites display an upregulation of panacelylated histone H3 and (C) panacelylated histone H4. The qChIP values are expressed as % of input and normalized for total histone H3 (B–C). (D) As in A–C, with he immunoprecipitation performed with a no-antibody control. The box plots on the right show the fold change distribution for each modified residue (B, C), or for Myc (A) in the two subpopulations (Non-targets and Targets). A two-sample t-test between the two subpopulations was performed and the relative p-value calculated (value within each box plot). Two-sample paired t-test was performed between (+Tet) and (8h) samples, for the two subpopulations and the p-value calculated (values at the bottom of each box plot).

**Figure 2 pone-0003650-g002:**
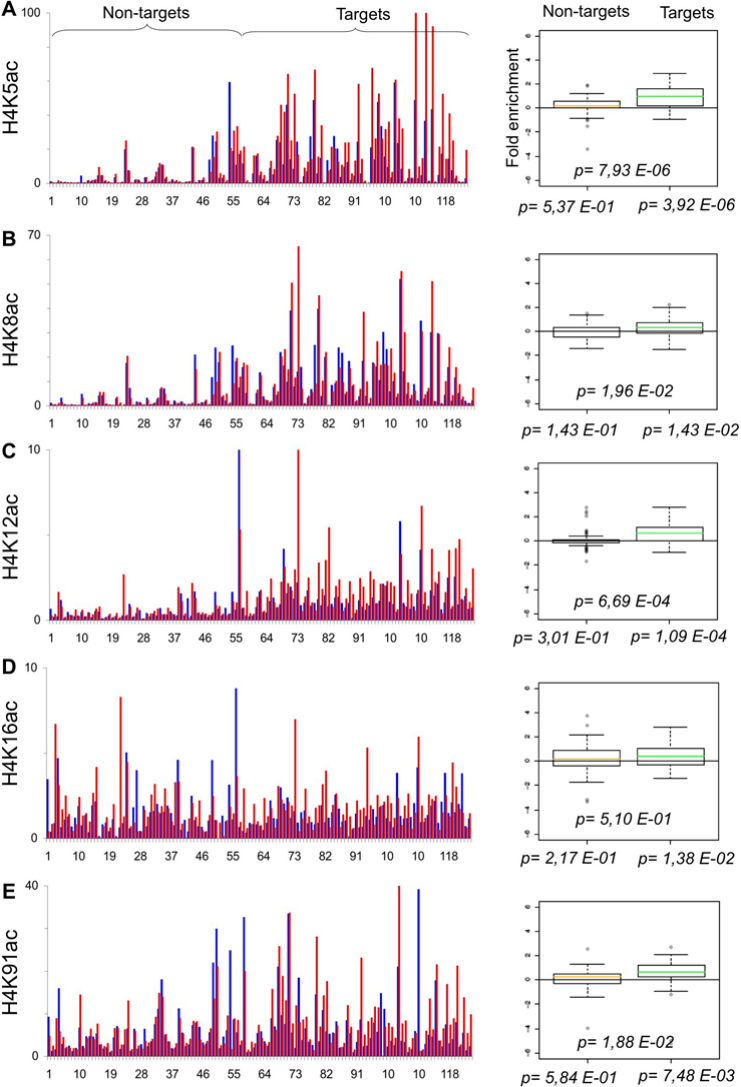
Myc regulates the levels of histone H4 acetylation. As in Fig 1. qChIP was performed using specific antibodies recognizing A. H4K5ac, B. H4K8ac, C. H4K12ac D. H4K16ac E. H4K91ac.

**Figure 3 pone-0003650-g003:**
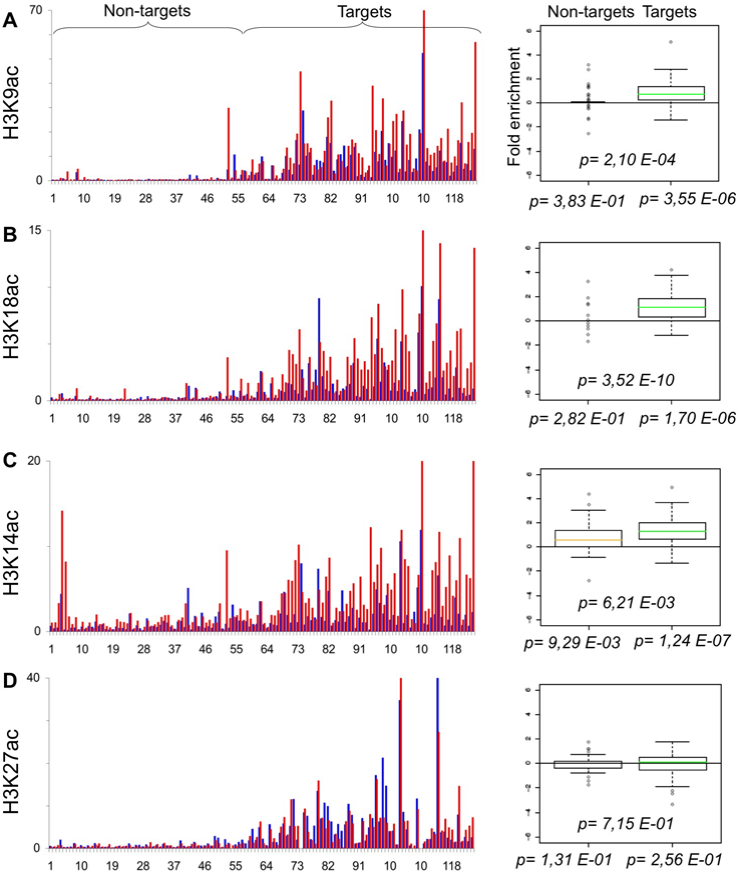
Myc regulates the levels of histone H3 acetylation. As in Fig 1. qChIP was performed using specific antibodies recognizing A. H3K9ac, B. H3K18ac, C. H3K14ac D. H3K27ac.

**Figure 4 pone-0003650-g004:**
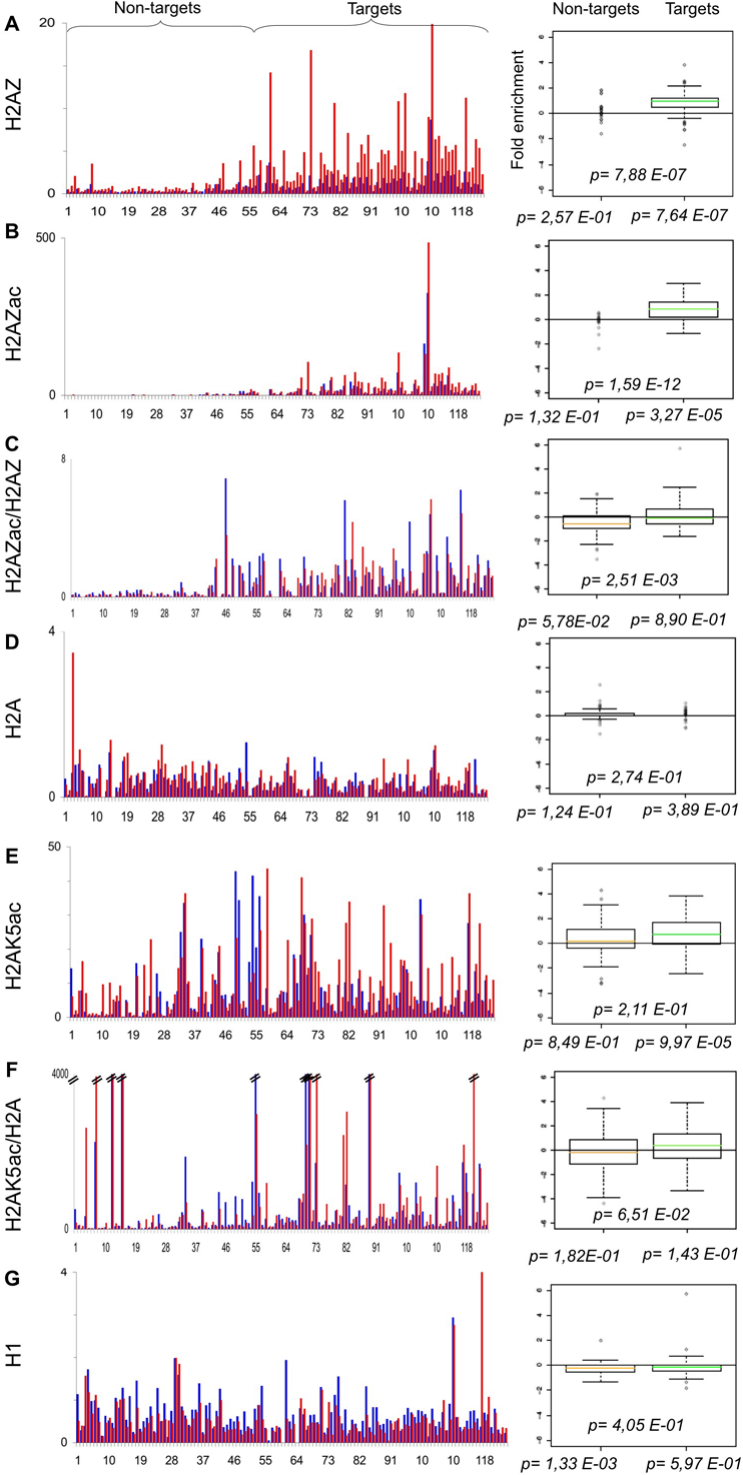
Myc affects the incorporation levels of histone variant H2A.Z. As in Fig 1. qChIP was performed using specific antibodies recognizing A. H2A.Z, B. H2A.Zac, D. H2A, E. H2AK5ac and G. H1. All the qChIP values are expressed as % of input and normalized for total histone H3, with the exception of C and F, where H2A.Z acetylation is noramlized for H2A.Z density, and H2AK5 acetylation is normalized for H2A density, respectively. The box plots show the fold change distribution of each acetylated residue for the two subpopulations. Statistical significance is calculated as in Fig. 1.

**Figure 5 pone-0003650-g005:**
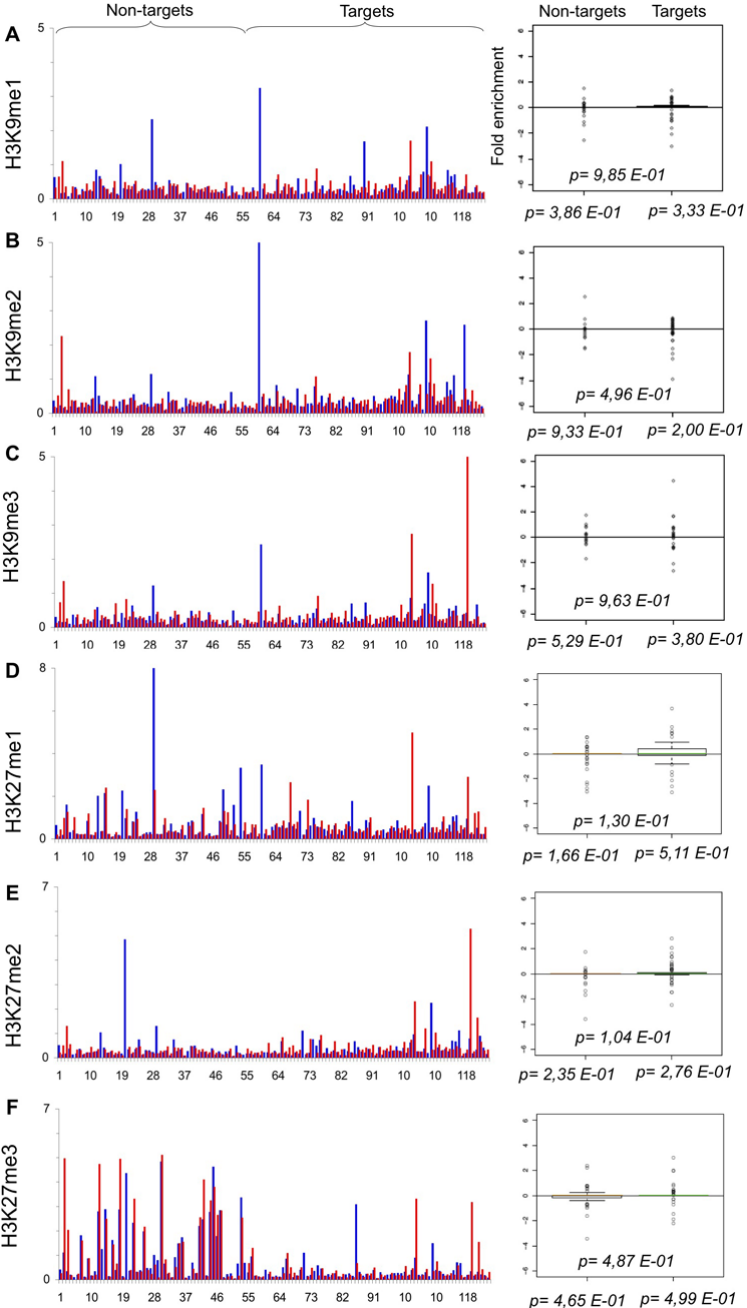
Myc does not alter the levels of heterochromatic marks. As in Fig 1. qChIP was performed using specific antibodies recognizing A–C. H3K9me1-me2-me3 and D–F. H3K27me1-me2-me3.

**Figure 6:**
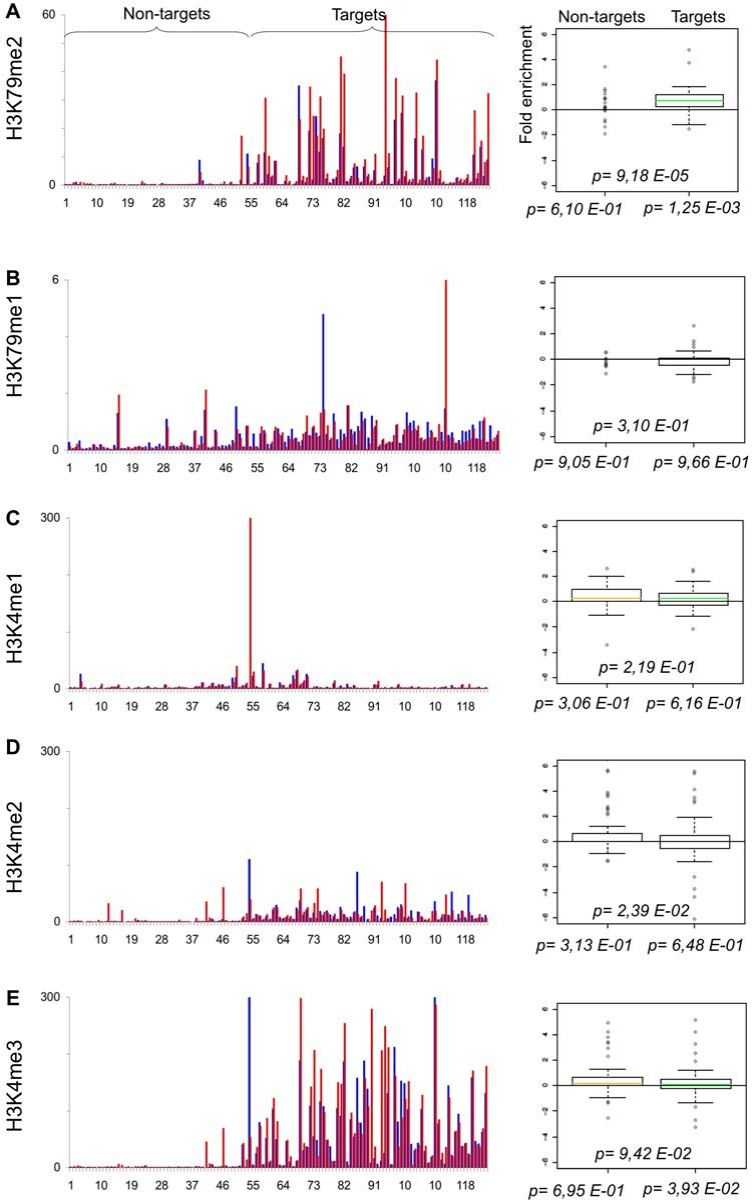
Myc regulates histone methylation in a promoter specific manner. As in Fig. 1, qChIP was performed using specific antibodies recognizing A. H3K79me2, B. H3K79me1, C. H3K4me1, D. H3K4me2 and E. H3K4me3.

**Figure 7:**
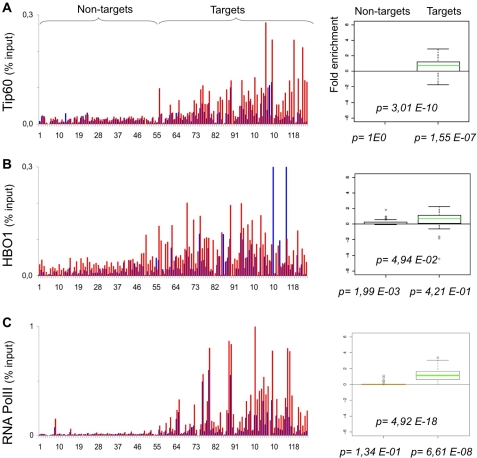
Analysis of Tip60, HBO1 and RNA PolII recruitment. As in Fig. 1, qChIP was performed using specific antibodies recognizing A. Tip60, B. HBO1, C. total RNA PolII.

The B-cell line P493-6 (or P493) expresses a Tetracycline (Tet)-repressible c-*myc* transgene [33]. We previously used these cells to profile Myc binding to E-box containing promoters by qChIP analysis [18]. We also profiled histone marks in the absence of Myc (+Tet) on two balanced populations of “Target” and “Non-target” promoters (originally dubbed “high-” and “low-affinity”, respectively) [25]. Here, we use the same system to profile the chromatin modifications induced by Myc: P493 cells were fixed 8 hours after Tetracyclin removal (−Tet), at which time Myc binding to chromatin was maximal [18], chromatin was immunoprecipitated with either of 27 antibodies, and the recovered DNA was used to amplify 124 different promoter sequences by real-time PCR. The recovery of each promoter sequence in each ChIP (expressed as % of input chromatin) is given in Supplementary Table S1. To account for nucleosomal density at any given site, the qChIP values for histone marks were then normalized to those of total histone H3. The resulting profiles along all promoters are displayed in Figures 1, 2, 3, 4, 5, 6. We also obtained qChIP profiles for two HATs (Tip60 and HBOI) and for RNA Polymerase II (RNA PolII), shown in Figure 7. All figures are organised as follows: the bar graph on the left reports the enrichment at every promoter either without (+Tet: blue bars) or with Myc (−Tet: red bars). Note that qChIP values are expressed as % of input chromatin for non-histone proteins, while histone marks or histone variants are normalized to total H3. Promoters (#1–124) are ordered along the X-axis by increasing Myc binding, shown in Fig. 1A, with full identification of all promoters provided in Table S1. This display allows distinction of Non-targets (#1–54) and Targets (#55–124), the two groups used statistical analysis. The box plot on the right displays the fold-change in qChIP value following Myc induction, averaged for the two different promoter groups. This allows us to report two fundamental parameters: first, whether each feature (Myc enrichment; fold-change for every histone mark) shows a significant change upon Myc induction within either promoter group (p-values shown below the X-axis); second, whether the two promoter groups are significantly distinct from one-another (p-value above the X-axis). As an example, we have aligned Myc binding with “pan” acetylation of histone H3 and H4: as previously reported, these marks are selectively induced on Myc-target genes [18], [26] (Fig. 1B, C).

We then examined the acetylation state of specific lysines on histones H4 and H3 (Fig. 2, 3). Myc most significantly enhanced H3K14ac, H3K18ac, H3K9ac, H4K5ac and H4K12ac on its Target promoters, followed by H4K8ac and H4K91ac. With the exception of H3K14ac, which showed moderate induction also on Non-target promoters (Fig. 3C), these marks were induced only in the Target group: this is consistent with the notion that Myc interacts with various HATs and recruits them to chromatin. Other acetylation marks were not, or less significantly regulated by Myc: H4K16 was modestly induced in both groups of promoters (Fig. 2D), as well as at several other locations far from a TSS (data not shown). H3K27ac was present selectively in the Target group [25] but, with the exception of few promoters, was not modulated by Myc. Of notice, in particular for H4, individual lysines did not account for the more significant level of regulation observed with the pan-acetyl antibody (Fig. 1B): indeed, individual marks were not induced on all targets and showed different patterns across the promoter population.

### Myc induces incorporation of the histone variant H2A.Z in target promoters

We previously identified histone H2A.Z as a component of euchromatic promoters in P493 cells [25]. In yeast, H2A.Z preferentially localizes in the two nucleosomes flanking the TSS [34]. In human cells, H2A.Z preferentially occupies the −1 nucleosome and is evicted from chromatin upon transcriptional activation [35], confirming the intrinsic instability observed in vitro [36]. Upon Myc induction, H2A.Z was consistently up-regulated on Target promoters, while no or little change was observed on non-target promoters (Fig. 4A). We observed a similarly selective increase in acetylated H2A.Z (Fig. 4B: H2A.Zac includes acetyl-K4, K7 and K11). Normalization of H2A.Zac to total H2A.Z showed that acetylation was higher at Target genes, but was not modulated by Myc itself (Fig. 4C). As a control, we also analysed total and K5-acetylated H2A: unlike what observed for H2A.Z, Myc did not induce H2A incorporation (Fig. 4D), although it enhanced its acetylation on a subset of promoters in either group (Fig. 4E, F). We conclude that Myc selectively induces H2A.Z incorporation in its target promoters (probably at the −1 nucleosome) [35], but does so without modifying its acetylation rate.

### Analysis of histone H3 lysine methylation

We next sought to analyse changes in methylation of specific H3 lysines. Methylation of H3K9 or H3K27 is associated with transcriptional repression and is generally low on Myc-target genes [25]. Indeed, we did not observe any significant changes in mono-, di- or tri-methylation of these residues upon Myc induction in P493 cells (Fig. 5).

Di-methylation of H3K79 (H3K79me2) is associated with transcriptional activity in human, fly and yeast cells [25], [37], [38], and specifically with productive transcriptional elongation by PolII [39]. Indeed, H3K79me2, but not H3K79me1, was up-regulated selectively at a subset of target promoters (Fig. 6A, B).

Di- and trimethylation of H3K4 are associated with active or poised promoters [3] and are present on Target promoters prior to Myc binding [25]. Myc activation had no general effect on either form of H3K4 methylation (me1, 2 or 3; Fig. 6C–E) although individual promoters did show up- or down-regulation. Significantly, in this context, Myc interacts with proteins regulating either methylation or demethylation of H3K4 (see Discussion).

### Chromatin modification patterns define different groups of Myc targets

**Figure 8:**
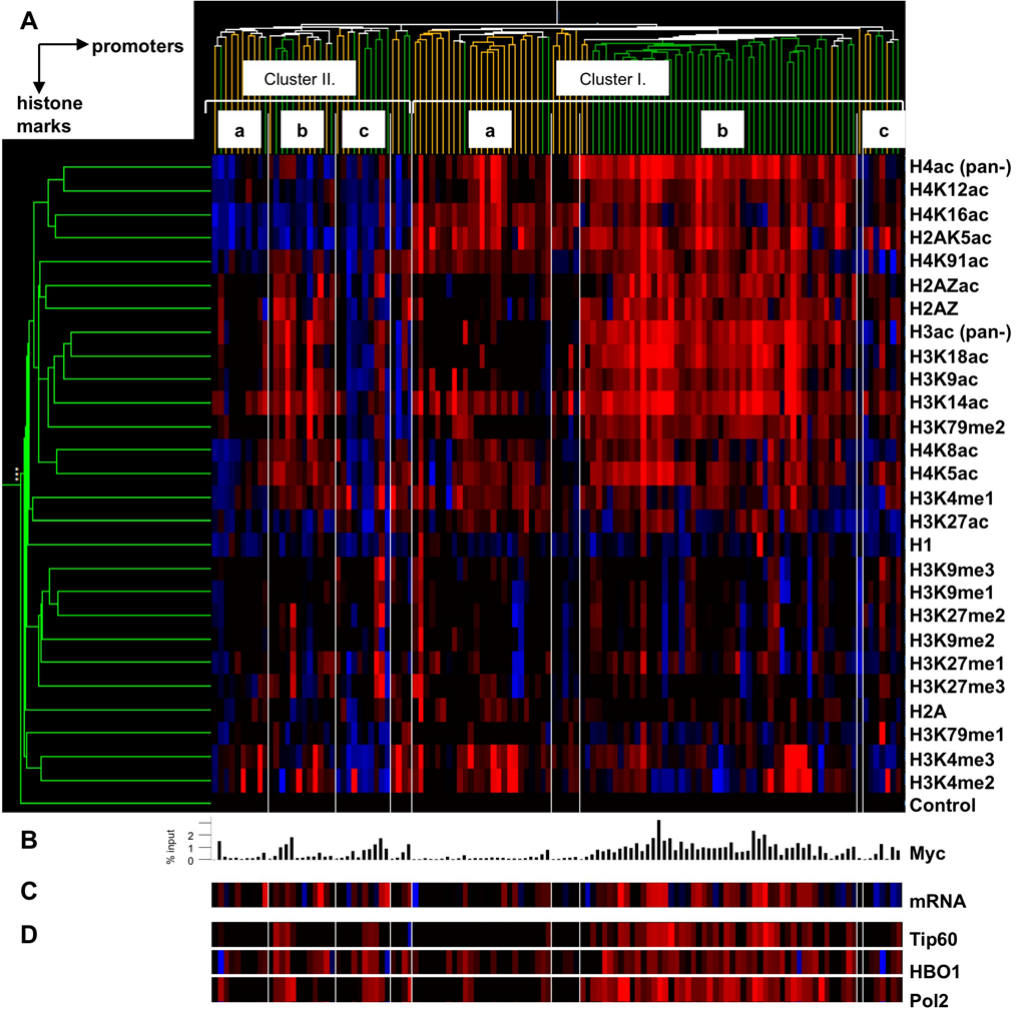
Clustering analysis of histone marks changes on human promoters. A. Quantitative chromatin immunoprecipitation (qChIP) was performed using 27 antibodies indicated on the right, plus a no-antibody control. The immunoprecipitations were performed with chromatin from p493 cells in the absence of Myc (+tetracycline, Tet), and 8 hours after Myc induction (−Tet). For this clustering analysis, the qChIP values for all histone marks on 124 promoters were normalized to those of total histone H3 and expressed as fold change values. B. Histogram representing Myc binding levels, reported as % of input. C. The expression level of each gene is represented as fold change upon Myc induction. D. qChIP was performed against Tip60, HBO1 and total RNA PolII. The ordering of the amplicons in B–D is based on the cluster analysis in A. The green and orange labels associated to each promoter indicate whether this is classified as Target (high-affinity) or Non-target (low-affinity) in this study.

The data presented so far indicated that Myc modulates acetylation and methylation marks to different extents, in different combinations and on different subsets of target promoters. The key question at this stage was whether these Myc-induced changes might identify different groups (hereby, clusters) of target loci, perhaps subject to different regulatory responses. To address this question, the above qChIP data were subjected to an unbiased clustering analysis, based on the variation (fold-change) of each histone mark on each promoter upon Myc induction (Fig. 8A). This was aligned with Myc binding levels at each locus (Fig. 8B), the fold-changes in gene expression (Fig. 8C), as well as in binding Tip60, HBOI and RNA PolII (Fig. 8D). Note that the parameters in Fig. 8B–D were not used for the clustering analysis, which was driven only by the changes in chromatin state: thus, their enrichment in any given cluster reflects their independent correlation with chromatin changes.

Based on this analysis, we identify two main clusters (I. and II.), distinguished mainly through the opposite regulation of H2AK5, H4K16ac, as well as H4K12ac (Fig. 8A, top 4 rows). As described above, H2AK5, H4K16ac did not systematically distinguish Target and Non-target promoters: logically, therefore, cluster I. and II. contained promoters of both types (Fig. 8A, B). The majority of Target and Non-target promoters segregated into two sub-clusters within cluster I. (I.a and I.b). I.a contained mainly Non-target promoters that showed increases in some acetylation marks (e.g. H4K16ac, H3K14ac, H2AK5ac and H4K91ac), but no significant induction of the main Myc-responsive marks (e.g. H3K9ac, H3K18ac, H2A.Z), no mRNA induction, and no loading of Tip60, HBOI or RNA PolII. Sub-cluster I.b, instead, contained promoters at which Myc consistently induced most, if not all of the responsive marks (H3K9ac, H3K18ac, H3K14ac H3K79me2, H4K12ac, H4K5ac, H4K8ac, H2AK5ac and acetylated H2A.Z loading) (Fig. 8A). Most of these loci were bound (Fig. 8B) and induced by Myc (Fig. 8C), and were those that most consistently showed loading of Tip60, HBOI and RNA Pol2 (Fig. 7, 8D). Collectively, these features identify the changes introduced by Myc on most of its direct target genes.

Other sub-clusters (I.c, II.a, II.b, II.c) contained smaller groups of loci, with different combinations of Target/Non-target genes and regulatory events, the significance of which remains to be addressed. Also, as noted previously [25], a minority of the E box-containing Target loci included in our study were repressed by Myc (in particular but not only within sub-cluster I.c). Owing to the small number of repressed genes covered, our data do not allow us to identify histone modifications that uniquely characterize those genes, and it remains to be addressed whether Myc can directly participate in transcriptional repression via E-boxes. Our data are consistent with the notion that Myc, when bound to this particular consensus, participates mainly to transcriptional activation.

## Discussion

### Myc regulates multiple acetylation events

Through the use of quantitative ChIP, we have recorded the changes induced by Myc in 25 histone marks on a representative collection of E-box (CACGTG)-containing promoters in the human B-cell line P493. Acetylation of several lysines (H3K9, H3K18, H4K5, H4K8, H4K12, H3K14 and H2AK5) was up-regulated at the majority of Myc-target promoters. The same acetyl-lysines in H3/H4 (except H3K14ac) are part of a “backbone” of 17 marks associated with active promoters at a genome-wide level in human T-cells [40] and, in yeast H4, have a non-specific, cumulative role in transcriptional activation [11]. Consistent with these observations, Myc-enhanced acetylation correlated with mRNA induction at the same target loci, suggesting that these marks might collectively contribute to transcriptional activation. Our data are also consistent with the notion that Myc interacts with multiple HATs and recruits these enzymes to its binding sites in chromatin, thereby enhancing histone acetylation: we confirmed the recruitment of Tip60 [26] and demonstrated that of the related enzyme HBO1, both of which preferentially acetylate histone H4 [41]. Myc binds other HATs, in particular GCN5/PCAF and p300/CBP [27], [28], [29], [42], [43], which are likely to contribute to histone acetylation at target promoters. The role of individual HATs in Myc-induced transcription remains to be addressed: we foresee that this will be significantly complicated by functional redundancy, both among HATs and among the different acetylated lysines. In this context, it is noteworthy that there was also significant heterogeneity in the exact combination of residues acetylated on different target promoters (Fig. 2–4; 8).

Another acetylation mark, H3K27ac was present selectively in the Target group but was not regulated by Myc. H3K27ac was also one of the 17 marks associated with active promoters throughout the genome [40], and is thus one of the euchromatic features that characterize target genes prior to Myc binding [25].

Of the aforementioned marks, only H3K14ac was induced by Myc also on Non-target promoters (Fig. 3C, 8), although less than in the Target group, implying that Myc had an additional effect on H3K14ac that did not depend upon direct DNA binding. Myc also caused a moderate, general increase of H4K16ac on both target and non-target promoters (Fig. 2D). This may be partly explained by the findings that H3K14 and H4K16 can be acetylated by GCN5 [44], and that GCN5 is encoded by a Myc-target gene, induced in P493 cells [45] (unpublished data). It should be noted, however, that GCN5 also targets H3K9 and H3K18 [46], [47], which were not acetylated at Non-target loci upon Myc induction (Fig. 2, 3, 8). Unlike other H4 lysines, H4K16 has a non-redundant function in transcriptional activation in yeast [11] and a unique role in counteracting compaction of the chromatin fibre [48]. This type of general regulatory mechanism may explain the role of Myc in maintaining bulk acetylation levels and preventing chromatin compaction [45].

### Myc induces incorporation of H2A.Z

Besides histone acetylation, Myc selectively induced incorporation of H2A.Z in its target promoters. Most interestingly, H2A.Z was one of the features associated with active promoters at the genome-wide level in human T-cells [40] and correlated with selective loss of the nucleosome at the −1 position [35], most likely required to accommodate the transcriptional apparatus at the promoter [49]. Whether the −1 nucleosome is evicted from Myc-target promoters and whether this occurs before or after Myc induction remains to be addressed. At most of target loci, however, RNA PolII appears to be loaded prior to Myc, although its levels consistently increase [25], [50] (Fig. 7C; see below).

The exchange of H2A.Z-H2B dimers is catalysed by the ATP-dependent chromatin-remodelling enzymes Swr1 in yeast, and by the homologous enzymes p400 or SRCAP in human cells [51], [52], [53], [54]. In yeast, the Swr1 complex shares several subunits with the HAT complex NuA4. The catalytic subunit of NuA4 is Esa1p, the yeast homologue of human Tip60 [55], [56]. In higher eukaryotes, the SWR1 and NuA4 complexes do not exist separately but are instead merged into the Tip60 complex [56], [57], which includes Tip60 as the HAT and p400 as the remodelling subunit [58], [59]. In all systems investigated, acetylation of H4 or H2A isoforms by Esa1/Tip60 seems to be closely connected with H2A.Z incorporation by Swr1/p400 [57], [60], [61]. Based on the observation that Myc recruited 5 subunits of the Tip60 complex (Tip60, p400, TRRAP, Tip48 and Tip49) to target promoters [26] we proposed that a similar mechanism may operate in Myc-induced transcription [62]. The data reported here lend further support to this model, by showing that recruitment of the complex (as marked by Tip60) is accompanied by incorporation of H2A.Z.

It should be noted here that a different hierarchical relationship between Tip60, p400 and H2A.Z has been implicated in the regulation of p53 target genes. At the p21 promoter, p53 was required for localization of p400 and H2A.Z at its distal binding sites prior to activation of the gene: upon activation of p53 signaling (by DNA damaging agents) p400 and H2A.Z were evicted, which occurred before Tip60 recruitment and gene activation [53]. Knockdown of either p400 or H2A.Z induced transcription of the p21 gene, which was prevented by Tip60 knockdown [53], [63], [64]. These observations may owe to the existence of p400 complexes that do not contain Tip60 [59], although the form of Tip60 recruited without p400 still has to be characterized. Altogether, it remains possible that Tip60 and p400 also exist in separate smaller complexes, as is the case in yeast.

Myc is also known to repress the p21 promoter via its association with the transcription factor Miz-1, located at the TSS [17], [65]. Most interestingly, Myc induced enrichment of both p400 and H2A.Z the p21 TSS [53], suggesting that these proteins also mediate transcriptional repression by Myc.

A recent genome-wide study in P493 cells showed that a significant fraction of Myc-target genes are repressed, which includes genes bound via canonical E-boxes [20]. This warrants further analysis of the mechanisms underlying repression by Myc through the E-box, besides the INR/Miz1 site. Among the E box-containing promoters in our collection, only a minority were repressed by Myc [25] (Fig. 8): most likely owing to this under-representation, our analysis of chromatin modifications has not led to the identification of a distinct pattern associated with transcriptional repression, and no definitive conclusions should be drawn in this regard.

### Effects of Myc on RNA PolII loading and elongation

Myc interacts with cyclin T1 and CDK9 (or pTEFb) and is thereby thought to promote Serine 2 (S2) phosphorylation in the PolII C-terminal domain (CTD) and transcription elongation [66]. Prior to S2, the CTD is phosphorylated on S5 by CDK7 (or TFIIH), marking promoters that are active or poised for activation [67], [68]. The effects of Myc on S2 and S5 phosphorylation remain to be systematically analysed by ChIP, and our efforts in this regard have remained inconclusive. However, loading of RNA PolII was clearly shown to precede Myc binding on multiple target promoters and in different cell types [25], [31], [32], [50], [66] (Fig. 7C). On several promoters targeted by both Myc and FoxO transcription factors, loading of PolII and the basal transcription machinery was Myc-independent, but instead required negative control of FoxO by the PI3K pathway; Myc recruited both TFIIH and pTEFb, thus controlling transcriptional elongation [50]. On the same promoters, Myc also promoted histone acetylation [31], [32]. Altogether, the available data do not support the notion that Myc regulates RNA PolII phsophorylation and histone acetylation on two different categories of genes [66]. Instead, the two mechanisms co-exist on the same promoters: most importantly, their functional relationship remains to be unravelled.

### Effects of Myc on histone H3 lysine methylation

As opposed to Myc-induced acetylation, little was know about potential effects of Myc on histone methylation. We have identified H3K79me2 as one of the marks preferentially induced by Myc on target promoters, concomitant with transcriptional activation (Fig. 6A, 8). Methylation of H3K79, catalyzed by the methyl-transferase Dot1L, has been associated with transcription elongation [39], [69]. Whether Myc directly regulates H3K79 methylation by recruiting Dot1L remains to be addressed, although we failed to detect an interaction between those proteins.

H3K4me2 and H3K4me3 mark transcriptionally active or poised promoters [3] and are a characteristic feature of Myc-target chromatin prior to– and independently from Myc binding [25]. Myc had no general effect on these methylation marks, although it did cause their up- or down-regulation on individual promoters (Fig. 6D, E). Significantly, in this context, Myc interacts with proteins regulating either methylation or demethylation of H3K4. First, Myc binds JARID-family demethylases [70] (our unpublished results). This interaction has been proposed to antagonize the demethylase activity of LID, the JARID1a-d homolog in Drosophila [70]. Second, Myc interacts with H3K4 methyl-transferases complexes of the MLL family (B. Lüscher, personal communication; our unpublished results). This association might serve a dual role. First, it may help targeting Myc to the appropriate chromatin environment, which precedes sequence-specific DNA binding. Such a mechanism is important for target gene recognition by Myc [25], as well as FoxA1 [71]. Second, Myc may contribute to recruit and/or stabilize MLL complexes at certain promoters.

Altogether, given the pre-existence of H3K4me3 and its independence upon Myc [25], the recruitment of either MLL- or JARID-family proteins is unlikely to be a general mechanism in Myc-regulated transcription, but might be relevant on promoters at which Myc up- or down-modulates H3K4me3. While there is clearly no obligate link between transcriptional activation by Myc and up-regulation of H3K4me3 (Fig. 8), the possible role of H3K4 de-methylation in repression remains to be addressed. We finally note that on/off and off/on transitions in Myc expression (either as achieved here with Tet-mediated regulation or in other instances with serum deprivation/stimulation) are adequate to study dynamic chromatin regulation by Myc, but will most likely miss its effects in epigenetic reprogramming. Besides being a potent oncogene, Myc is one of four factors that, when co-expressed, contributes to reprogram somatic cells to a pluripotent state [72], [73], [74]. Other studies have associated Myc with self-renewal of Embryonic Stem (ES) cells [75] and with the induction of an ES-like gene expression profile is a variety of cells and tumors [76]. These findings warrant further studies on the control of histone methylation by Myc during cellular transformation and reprogramming.

## Methods

### Cell culture

P493-6 cells were grown in suspension in RPMI supplemented with 10% foetal calf serum (FCS), NEAA (BioWhittaker), L-glutammine and antibiotics. For ChIP, 2 L of logarithmically growing cells were split to 3×10^5^ cells/ml, and 0.1 mg/ml tetracycline (Sigma) was added for 72 h. To reinduced expression of Myc, cells were washed three times with PBS and then cultured for 8 h (RPMI).

### Antibodies

The antibodies used for ChIP were the following. From Santa Cruz: c-Myc (sc-764), PolII (sc-899). From AbCam: ab18255 H2A; ab18262 H2A.Zac; ab7789 H1; ab1764 H2AK5ac; ab4174 H2A.Z; ab1191 H3K18ac; ab4441 H3K9ac; ab7766 H3K4me2; ab3594 H3K79me2; ab8895 H3K4me1; ab2886 H3K79me1; ab7312 H3K9me2; ab8580 H3K4me3; ab1791 H3; ab1761 H4K12ac; ab1762 H4K16ac; ab1758 H4K5ac; ab1760 H4K8ac; ab4627 H4K91ac. From Upstate: 06-866 H4pan-ac; 06-599 H3pan-ac; 07-327 H4K5ac; 07-328 H4K8ac; 07-323 H4K12ac; 07-329 H4K16ac; 07-360 H3K27ac; 07-354 H3K18ac; 07-353 H3K14ac; 07-473 H3K4me3. H3K9me1, me2, me3 and H3K27me1, me2, me3 were kindly provided by T. Jenuwein [77]. Anti-Tip60 rabbit polyclonals have been previously described [26], [78]. The anti-HBO1 polyclonal has been raised immunizing rabbits with a C-terminal peptide of human HBO1.

### Quantitative Chromatin Immunoprecipitation (qChIP)

P493-6 cells were processed for qChIP following our original protocol with further modifications. Formaldehyde (37%) was added to the culture medium to a final concentration of 1%. Cross-linking was allowed to continue for 10 min at room temperature and stopped by addition of glycine (0,125 M as final concentration), followed by an additional incubation of 5 min. Fixed cells were washed twice with PBS and harvested in SDS buffer (50 mM Tris at pH 8.1, 0,5% SDS, 100 mM NaCl, 5 mM EDTA, and protease inhibitors). Cells were pelleted by centrifugation, and resuspended in 3 ml of IP buffer (100 mM Tris at pH 8.6, 0.3% SDS, 1.7% Triton X-100, and 5 mM EDTA). Cells were disrupted by 5–7 pulses (30 sec each one) of sonication with a tapered ¼-microtip (6,5 mm) in a Branson digital sonifier 250 D, at a power setting of 30%, yielding genomic DNA fragments with a bulk size of 300–500 bp. For each immunoprecipitation, 1 ml of diluted lysate (5×10^7^ cells/ml) was precleared by addition of 300 µl blocked protein A or G beads (50% slurry protein A-Sepharose from Amersham or Rec-protein G-Sepharose 4B ZYMED from invitrogen; 0.5 mg/ml fatty acid free BSA, Sigma; and 0.2 mg/ml salmon sperm DNA in TE). Samples were immunoprecipitated overnight at 4°C with antibodies specific for factors or modified histones. Immune complexes were recovered by adding 50 µl of bloked beads and incubated for 2 h at 4°C. Beads were washed and crosslink was reversed in 1% SDS and 0.1 M NaHCO3. The eluted material was purified with QIAquick PCR purification Kit, Qiagen. The obtained DNA was resuspended in 1ml of T buffer (Tris HCl 10 mM pH 8) and used directly for qPCR.

### Quantitative PCR

Real-time PCR was performed with 6 µL of DNA per reaction and 200 nM primers, diluted in a final volume of 20 µL in SYBR Green Reaction Mix (Perkin Elmer, Boston, MA). Accumulation of fluorescent products was monitored by real-time PCR using a GeneAmp 9700 Sequence Detector (ABI). Each PCR reaction generated only the expected specific amplicon, as shown by the melting-temperature profiles of final products (dissociation curve, automatically measured by the Taqman 9700). No PCR products were observed in the absence of template. PCR primer sequences are given in Supplementary Information (Table S1).

### Bioinformatic analysis

Data analysis was performed using R 2.6.0 and GeneSpring GX 7.3.1 (Agilent Technologies). For the clustering of Figure 8 and the boxplots of Figures 1–6 DNA recovery values (percentage of input DNA) for each histone ChIP were normalized to those of total H3 for the 124 sites analysed. A threshold for low raw values was applied (0.39 for histone marks, 0.032 for transcription factors/co-factors; the threshold were calculated based on the highest values of the control/negative ChIPs), and fold change between −Tet/+Tet was calculated. Hierarchical clustering was generated by computing distance based on Sperman's rank (GeneSpring implemented algorithm) and using an average linkage method. Fold change was log transformed and boxplots were created using R. Two-sample t-test between Myc-targets and non-targets was performed and the relative p-value calculated. Two-sample paired t-test was performed between (+Tet) and (−Tet) samples and the p-value calculated.

## Supporting Information

Table S1 Genomic coordinates, PCR primers and qChIP data. The columns are defined by the following headings: Amplicon#: Each PCR amplicons is identified by the reference number of its 5′ primer in our laboratory database. X-axis Fig. 1–6: Numbering of each locus/amplicon on the X-axis of all bar-graphs in figures 1–6. These numbers correspond to increasing Myc binding, determined by the qChIP values in column Myc (−tet). Order Fig. 8: The number indicated the order of appearance of each locus in the clustering analysis of Fig. 8. Chr, Start, End: chromosomal coordinates of each PCR amplicon. Forward, Reverse: sequences of the corresponding PCR primers. Amplicon Seq, Length: sequence and length of each amplicon. Gene: name of the gene. For bi-directional promoters, the two genes are named. mRNA: Variation in mRNA levels for each gene. For bi-directional promoters, the data refer to the first gene listed. Data are represented as the ratio of mRNA levels in the two experimental conditions (−/+tet) normalized to 18S levels. ND: mRNA not detected (in either −tet or +tet). na: mRNA not addressed. All other columns: The numbers show qChIP data (expressed as % of input chromatin) for each protein or histone mark analyzed (indicated in the heading) with or without tetracylin (+tet, −tet).
(0.27 MB XLS)
